# Gender, Assets, and Agricultural Development: Lessons from Eight Projects

**DOI:** 10.1016/j.worlddev.2016.01.009

**Published:** 2016-07

**Authors:** Nancy L. Johnson, Chiara Kovarik, Ruth Meinzen-Dick, Jemimah Njuki, Agnes Quisumbing

**Affiliations:** a International Food Policy Research Institute (IFPRI), USA; b Canada’s International Development Research Centre, Nairobi, Kenya

**Keywords:** gender, assets, property rights, agriculture, impact evaluation

## Abstract

Ownership of assets is important for poverty reduction, and women’s control of assets is associated with positive development outcomes at the household and individual levels. This research was undertaken to provide guidance for agricultural development programs on how to incorporate gender and assets in the design, implementation, and evaluation of interventions. This paper synthesizes the findings of eight mixed-method evaluations of the impacts of agricultural development projects on individual and household assets in seven countries in Africa and South Asia. The results show that assets both affect and are affected by projects, indicating that it is both feasible and important to consider assets in the design, implementation, and evaluation of projects. All projects were associated with increases in asset levels and other benefits at the household level; however, only four projects documented significant, positive impacts on women’s ownership or control of some types of assets relative to a control group, and of those only one project provided evidence of a reduction in the gender asset gap. The quantitative and qualitative findings suggest ways that greater attention to gender and assets by researchers and development implementers could improve outcomes for women in future projects.

## Highlights

• We synthesize the findings of 8 impact evaluations of agricultural projects.
• Four projects increased some types of women’s individually-owned assets.
• Jointly-owned assets are a significant part of asset portfolios.
• Agricultural development projects should pay closer attention to asset ownership.

## 1 Introduction

For many years development interventions focused on increasing incomes to reduce poverty; now a growing body of evidence emphasizes the importance of assets for poverty reduction (Adato et al., 2006, Barrett and Swallow, 2006, Carter and Barrett, 2006, Carter et al., 2007, Carter and May, 2001, Jalan and Ravallion, 2002, Lybbert et al., 2004, Naschold, 2012, Naschold, 2013, Winters et al., 2009) as well as for individuals’ and households’ current and long-term well-being (Schreiner & Sherraden, 2007). A body of work also exists on the importance of women’s ownership of and control over assets for a range of development outcomes, both for women themselves and for their families (Haddad et al., 1997, Meinzen-Dick et al., 2011, Quisumbing and Maluccio, 2003). Yet, men are generally advantaged in owning assets, given the gender norms that govern asset ownership, which means that they tend to own more assets and assets of higher value than women (Deere and Doss, 2006, Deere et al., 2013, Quisumbing and Maluccio, 2003).

While building women’s assets has become a global development priority (Deere et al., 2013, FAO, 2011, Meinzen-Dick et al., 2011), few agricultural interventions consider their impacts on assets at the individual or even household level. To better understand the importance of gender and assets in agricultural development projects, and the potential of projects to build women’s assets, the Gender, Agriculture, and Assets Project (GAAP) worked with eight agricultural development projects in Africa and South Asia to build explicit attention to gender and gendered ownership of assets into their monitoring and evaluation plans. The eight projects, which took place in seven different countries, covered different types of interventions with different implementation approaches. They took diverse approaches to gender—ranging from gender blind to gender transformative—and to assets, with some projects distributing agricultural assets such as land, livestock, or machinery and others promoting increased productivity through access to inputs and training. In each project evaluation, both qualitative and quantitative methods were used to look at how participants understood gendered use, control, and ownership of assets; how assets influenced who was able to participate in and benefit from projects; and how projects impacted a range of outcome measures, including women’s access to and control over assets.

This paper synthesizes the findings of the project evaluations and related analyses from GAAP. Section 2 presents the GAAP conceptual framework, and Section 3 describes the eight projects and the key elements of their evaluation designs. Section 4 characterizes the gender norms and context in project countries using secondary data. Section 5 summarizes the findings of the evaluations on changes in use, control, and ownership of assets. Subsequent sections unpack the findings by looking at links between assets and key outcomes identified in the conceptual framework—livelihood strategies (Section 6), control of income (Section 7), and well-being (Section 8). Section 9 summarizes lessons for program implementers on how to incorporate gender and assets into program design, implementation, and evaluation. It also identifies areas where further research is needed to better understand how to define and measure gendered asset ownership.

## 2 The gender, agriculture, and assets project conceptual framework^1^

The term *asset* is often used very loosely in discussing resources that individuals, families, or other organizations (groups, corporations) control. Carter and Barrett (2006, p. 179) define assets as “conventional, privately held productive and financial wealth, as well as social, geographic, and market access positions that confer economic advantage.” The accounting definition of assets considers these as economic resources—“anything tangible or intangible that is capable of being owned or controlled to produce value and that is held to have positive economic value. Assets represent value of ownership that can be converted into cash (although cash itself is also considered an asset)” (Sullivan & Sheffrin, 2003, p. 272). In the international development literature, another way that assets are understood comes from the Sustainable Livelihoods framework (Scoones, 1998). This framework recognizes five capitals—natural (land, water), physical (agricultural and household durables), financial (cash or savings), human (health, knowledge, skills), and social (group membership, social networks)—and posits that these capitals underlie the ability of households to engage in livelihood strategies.

As suggested by the above definitions, a key part of the definition of an asset has to do with its ownership and control. Ownership is often understood simplistically as a binary variable; however, property rights over assets can be very complex, as suggested by the legal definition of property rights—the relationships among people over things (Cohen, 1954). Property rights are generally defined based on a person’s ability to use an asset for specific purposes or to make decisions about how it will be used by others. Ownership of an asset generally means possession of a “bundle of rights” over that asset. Schlager and Ostrom (1992) characterize different bundles of rights along a continuum from use rights to control rights to ownership rights. Examples of some use or access rights include the right to live in a house, to fish in a lake, or to milk a cow. Some control or decisionmaking rights include the right to decide who else lives in the house or fishes in the lake, what the cow eats, what crops to plant on a plot of land, and whether to exclude others from grazing their animals on a particular pasture (Meinzen-Dick, Pradhan, & Di Gregorio, 2004). Full ownership often includes all of these rights as well as the right to dispose of an asset (the house or the cow), whether through sale, lease, gift, or inheritance transfers.

**Figure 1:**
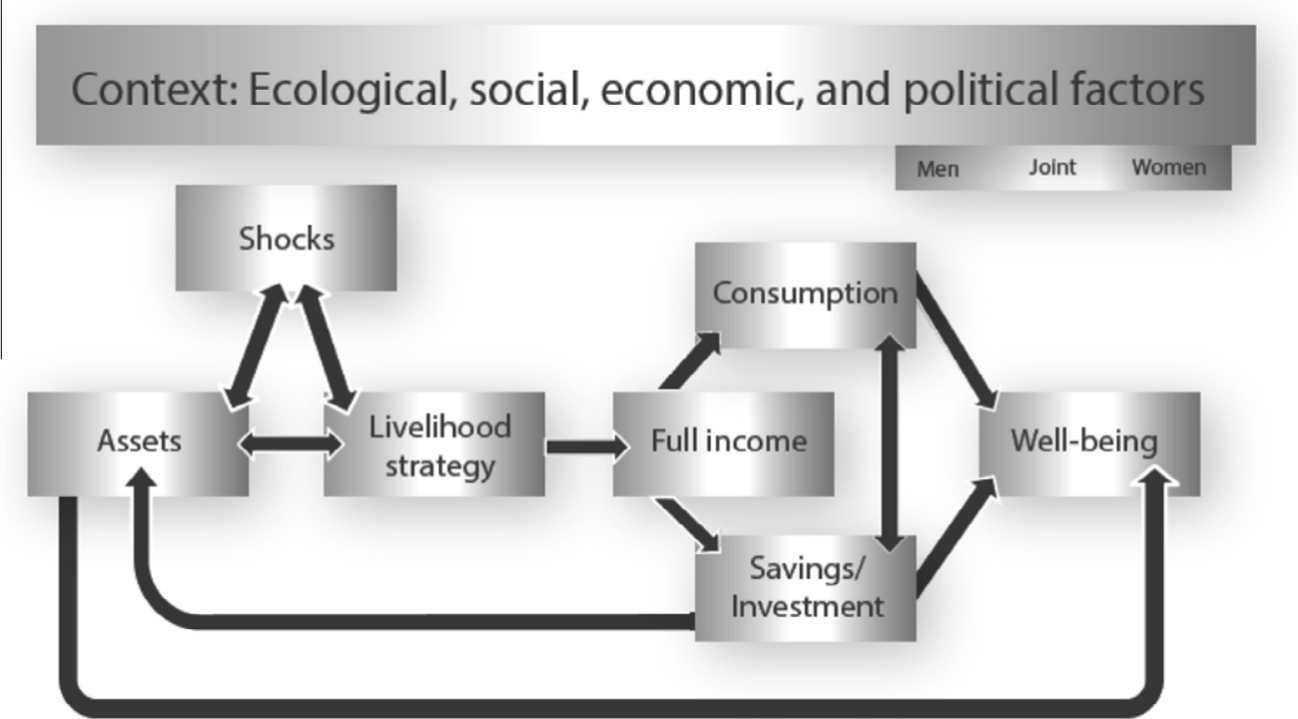
The Gender, agriculture, and assets conceptual framework. Source: Meinzen-Dick *et al.* (2011, p. 4).

The GAAP conceptual framework diagram (Meinzen-Dick *et al.*, 2011) provides an illustration of the relationships between gender, assets, and well-being in the context of agricultural development (Figure 1). The shading in the figure reflects the fact that components are gendered, meaning that they might be different for men than for women within a household. Households are important units of analysis in development programing; and many projects, including the majority of the projects in GAAP, define their beneficiaries as, and design their programs to target, households. Households are made up of individuals, however, and an intervention may affect different household members differently. It is important to take this into account to understand how an intervention is likely to work. This applies even to the context as certain social, economic, or political factors may affect women and men differently, while others affect a household as a whole.

Assets^2^ can influence the design, implementation, and outcomes of programs by determining who participates (and who does not participate) in the programs as well as how and how much they benefit. Some agricultural projects distribute agricultural assets such as land, livestock, infrastructure, or machinery. Agricultural interventions can also introduce improved technologies or institutional innovations that increase the returns to the productive assets used in agriculture-based livelihood strategies, potentially raising the returns to and value of some assets (and possibly lowering others) as well as producing surplus that can be reinvested in asset accumulation.

Although societal norms govern the gendered distribution of assets, it is by no means immutable. Agricultural development programs may shift the gendered asset distribution. This could happen directly through, for example, direct asset transfers to women, or training, perhaps in combination with efforts to influence attitudes. It can also happen indirectly through the downstream impacts of projects on gendered control of incomes and investment opportunities. These latter effects may be unintentional and may result in worse outcomes for women if their access to or control over assets is weakened. These are the dynamics that GAAP investigated in the context of these eight agricultural development projects.

## 3 The gender, agriculture, and assets project

### (a) Project portfolio

The GAAP was jointly led by the International Food Policy Research Institute (IFPRI) and the International Livestock Research Institute (ILRI) and was supported by the Bill & Melinda Gates Foundation from 2010 to 2014.^3^ GAAP’s goal was to better understand gender and asset dynamics in agricultural development programs. GAAP research team members from IFPRI and ILRI worked with eight agricultural development projects in Africa south of the Sahara and South Asia (Table 1) to identify how development projects impact men’s and women’s assets; to identify strategies that successfully build women’s assets and reduce gender gaps in asset access, control, and ownership; and to improve each participating organization’s abilities to measure and analyze qualitative and quantitative gender and asset data.

**Table 1:** The GAAP portfolio

Project Implementer	Project name	Country (ies)	Years	Project objectives	Implementation approach
BRAC	Challenging the Frontiers of Poverty Reduction – Targeting the Ultra Poor (CFPR-TUP)	Bangladesh	2007–11	To assist the ultra-poor in rural Bangladesh to graduate from ultra-poor status and access mainstream development programing	Provided small grants to female household members, and provided participating households with assets and intensive training on how to use the assets
Harvest Plus	Reaching End Users (REU) Orange Sweet Potato (OSP)	Uganda	2007–09	To increase vitamin A intake and reduce vitamin A deficiency among vulnerable populations (women and children)	OSP vines were disseminated through pre-existing farmers’ groups composed largely or entirely of women
Helen Keller International	Enhanced Homestead Food Production (E-HFP)	Burkina Faso	2009–12	To improve the nutritional status of infants, young children, and mothers through improved access to nutritious foods year-round and the adoption of optimal nutritional practices	Worked with mothers to establish homestead gardens. The project provided inputs and trainings in gardening, irrigation, and small livestock rearing to beneficiary women. Project also trained beneficiary women in improved nutrition practices using behavior change communications
Kickstart International	Treadle Pumps in East Africa	Kenya and Tanzania	2010–12	To enable poor farmers to move out of poverty through increased yields and crop production achieved through manually operated, low-cost, micro-irrigation treadle pumps	Used a market-based system of distributors to increase access to pumps
Landesa	Micro-land Titling for India’s Landless Agricultural Laborers	India	2010–15	To work with state governments and local communities to reduce poverty through regularization and titling of homestead land in Odisha and allocation and titling of homestead plots in West Bengal	Programs promoted inclusion of women’s names on land titles and promote land security for widows and other vulnerable groups. Program also provided a variety of forms of assistance for housing and basic inputs, capacity building in homestead food production, and promotion of local development of roads, water, and terrain leveling
Land O’Lakes	Manica Smallholder Dairy Development Program	Mozambique	2008–12	To rebuild Mozambique’s dairy industry to meet market demand, and to increase incomes for smallholder farmers by participating in a sustainable dairy value chain	Provided improved dairy cows and training to beneficiary households
CARE	Strengthening the Dairy Value Chain (SDVC)	Bangladesh	2007–12	To improve the dairy-related incomes of 35,000 smallholder farmers in northwest Bangladesh	Improved smallholder participation in the value chain, namely farmer mobilization and education, access to markets for their milk, and access to productivity-enhancing inputs. Assisted in the formation of dairy farmer groups, selection of farmer group leaders, selection of dairy collectors and livestock health workers, and training of all involved
International Rice Research Institute (IRRI)	Cereal Systems Initiative for South Asia (CSISA)	India	2009–11	To reduce food and income insecurity through accelerated development and deployment of new cereal varieties, sustainable crop and resource management practices, and better access to information	Included widespread delivery and adaptation of production and postharvest technologies as well as promotion of crop and resource management practices and high-yielding, stress-tolerant, and disease-and insect-resistant rice, wheat, and maize varieties and hybrids

Several criteria were used to determine project inclusion into the GAAP portfolio: inclusion of baseline and endline data collection in project activities; willingness of the projects to incorporate gender into their evaluation design; alignment of the project time frame with the GAAP time frame; and willingness of project teams and evaluation teams to invest time in training, information sharing and other GAAP activities. The alignment of project and GAAP time frames meant that projects would have already completed baselines, or be very close to completing them, at the time they joined GAAP. A consequence of this requirement was that, in most cases, the baseline data did not contain sufficient information on gendered use, control, and ownership of assets, and that this had to be built up during the course of the project, either through retrospective methods or through qualitative work.

While we were ultimately able to collect and use quantitative data on gendered ownership of key assets in seven of the eight project evaluations, the rigor and quality of the evaluation designs (Table 3) and the data were variable, in particular in terms of the ability to answer questions about the net effects of interventions on the total asset base rather than on individual assets or asset categories, and in terms of the ability to synthesize results across studies. This was largely because the studies were initiated separately to answer project-specific questions and evaluation needs, and because we were aiming for representativeness across different types of assets. Although the existing evaluation design was an important criterion in screening projects that applied to join GAAP, there was still substantial variability in the quality of the evaluation designs as well as organizational capacity to undertake the necessary analyses. Adding on the GAAP component, in particular the qualitative analysis, was a cost effective way to begin exploring how agricultural projects affect and are affected by the gendered distribution of assets. Comparing results across projects identifies key trends and common themes but perhaps raises more questions than it answers in terms of what works for women in agricultural development projects. These questions will be addressed in GAAP Phase 2 (2015–20), in which we will have a larger portfolio of projects selected specifically to facilitate comparative analysis and synthesis.

**Table 2:** Key gender and asset aspects of the projects

Project implementer	Country	Asset-related participation requirement	Main mode of building assets	Approach to gender at start of project
Landesa	India	Currently accessing a plot	Land transfer and regularization	Gender aware
BRAC	Bangladesh	None	Land and livestock transfer	Gender aware
CARE	Bangladesh	Cow	Increasing production and income	Gender transformative
Land O’Lakes	Mozambique	Land and cattle feed	Cow transfer	Gender blind
Helen Keller International	Burkina Faso	None at individual level	Land and tools transfer	Gender transformative
HarvestPlus	Uganda	Implicit requirement of land access	Increasing access to planting material	Gender aware
IRRI	India	Implicit requirement of land access	Increase awareness and availability of agricultural technologies	Gender blind
KickStart	Kenya and Tanzania	Implicit access to land and water	Marketing of pumps, education, and awareness building	Gender blind

*Source:* Authors.

*Notes:* The classification of the project approach is adapted from several sources including Manfre and Rubin (2012), Bill and Melinda Gates Foundation (2012), Caro (2009), and Rubin, Manfre, and Nichols Barrett (2009). *Gender blind* refers to efforts that “typically do not acknowledge the role of gender in different social contexts and ignore the different ways that men and women engage with productive resources.” *Gender aware* refers to approaches that “have an understanding of the different needs and interests of men and women.” *Gender transformative* refers to approaches that “explicitly engage both women and men to examine, question, and change those institutions and norms that reinforce gender inequalities.”

**Table 3:** Project evaluation design and GAAP contribution

Project implementer	Evaluation design	GAAP contribution
Landesa	Propensity-weighted regressions	Qualitative work (FGDs, KIIs, life histories); input into quantitative survey module
BRAC	Randomized controlled trial	Qualitative work; input into gender and assets modules in endline
CARE	Propensity-weighted regressions	Qualitative work; input into gender and assets modules, additional modules for endline
Land O’Lakes	Early *vs.* late cow recipients	Qualitative work (FGDs, KIIs, life histories); input into quantitative survey module
Helen Keller International	Randomized controlled trial	Qualitative work; input into gender and assets modules
HarvestPlus	Randomized controlled trial	Qualitative work, including social network analysis; input into gender and assets modules
Cereal Systems Initiative for South Asia	Comparator control villages	Qualitative and asset module in midline quantitative survey; funding for analysis time to focus on social networks
KickStart⁎	Early *vs.* late pump buyers	Funding for qualitative work

*Source:* Authors.

*Note:* FGDs = focus group discussions; KIIs = key informant interviews.

⁎ In KickStart, only qualitative results were used in the analysis.

### (b) Summary of asset-related aspects of project design

Table 2 summarizes key gender- and asset-related aspects of the design and implementation of the projects. In terms of their approaches to gender, nearly all projects worked with men and women within beneficiary households; however, only five (BRAC, HarvestPlus, HKI, Landesa, and CARE^4^) specifically targeted women as beneficiaries. Half of the projects (BRAC, HKI, Landesa, and Land O’Lakes) distributed assets, while HarvestPlus distributed vines through farmers’ groups. Nearly all projects built human or social capital in the form of training or group formation or strengthening. In most cases, GAAP did not attempt to measure impacts on human or social capital, although they are considered in the analysis of project implementation and impact. The exceptions were projects that focused on nutrition outcomes (HarvestPlus and HKI).

Nearly all projects also had asset-related participation requirements (Table 2). CARE and Land O’Lakes explicitly required that participants possess certain assets: a dairy cow in the former, and adequate access to land and water and initial fodder stocks in the latter. The KickStart and IRRI projects had implicit requirements based on access to land or water. Landesa’s project, by providing titles to land that households were already cultivating, also had an implicit land access requirement. HarvestPlus had an implicit social capital and potentially also a land requirement by distributing vines through farmer groups. Note that for many of the projects that required access to land, this access did not have to come through formal landownership. Unfortunately, failure to make that clear in some cases resulted in women not being considered as official beneficiaries.

### (c) Key elements of project evaluation designs

GAAP provided technical and financial support to enable projects to incorporate gender and assets into their existing evaluation plans. Beyond promotion of mixed methods, GAAP’s contribution to each project varied according to project needs and the project teams’ gender and monitoring and evaluation capacity. Table 3 summarizes each project’s evaluation approach and highlights GAAP’s contribution. GAAP’s financial contribution of approximately $100,000 per project constituted a small fraction of the total project budgets (which ranged from about $2 million to $195 million). GAAP’s contribution to the project’s monitoring and evaluation budget ranged from less than 1% to about 50% (Firetail Limited, 2014).

## 4 Contexts in which the gender, agriculture, and assets project operated

The eight projects in the GAAP portfolio operated across a wide variety of country contexts, with four projects in South Asia and four in Africa south of the Sahara. As suggested in Figure 1, understanding the contexts within which the projects operated is important to understanding their success and the differences in outcomes. Especially important is understanding social and gender norms and their implications for rural women’s ability to participate in and benefit from projects. Provided below are two tables that present data that reflect different aspects of women’s (Table 4) and men’s (Table 5) status in project countries, with data primarily drawn from the most recent set of Demographic and Health Surveys (DHS) Country Reports. Comparison between Table 4 and Table 5 allow for an understanding of the gender gaps that exist between men and women in the project countries.

**Table 4:** Women’s status in project countries

Variable	Countries						
	Bangladesh	India	Burkina Faso	Kenya	Tanzania	Mozambique	Uganda
Project implementers operating in country	BRAC, CARE	Landesa, IRRI	Helen Keller International	KickStart	KickStart	Land O’Lakes	Harvest Plus

Households headed by female (%)	11.0	14.9	8.3	35.8	24.8	35.3	29.2
Ever married (%) l^∗^	85.4	82.8	79.4	68.8	74.9	81.6	75.6^e^
Median age at first marriage ++	15.6	16.4	17.6^j^	19.4	18.5	18.2	17.8
Fertility rate	2.5	2.98	6.7	5.2	6.1	6.6	6.8
Median age at first birth ++	18.1	19.5	19.2^k^	19.9	19.5	19.2	18.9
Median years of schooling	4.0	NR	NR	4.5^f^	6.2	1.4	4.6
Literate (%)	59.7	45.5	11.4	82.3	66.1	25.5	58.8
Currently employed (%)	10.3	40.8	79.1	55.5	82.2	39.3	70.5
No access to information (%)^a^	58.6	45.4^g^	56.9	22.6	44.5	57.0	24.2
Involved in decisionmaking (%)^b^	56.5	48.9	17.6	65.5	36.7	54.4	56.5
Agree with wife beating (%)^d^	35.6	59.4	47.0	58.9	57.7	25.3	61.3
Landholders that are women (%)	2.8	10.9	8.4	NR	19.7	23.1	16.3

*Source:* Authors. See Appendix for data sources.

Notes: All figures refer to rural population unless otherwise noted by an asterisk (∗). NR = not reported. ++ = ages 20–49. a = the percentage of women that do not read the newspaper, listen to the radio, or watch television at least once a week. b = the percentage of women that are involved in decisionmaking, either alone or jointly with their husband, about major household purchases. d = the percentage of women that agree with at least one reason that a husband is justified to beat his wife. e = percentage includes “living together,” which comprises 26.9%. f = females age 6 and up. g = phrased slightly differently: “percentage of women not regularly exposed to any media.” j = women ages 25–49. k = this indicator refers to women ages 25–49. l = the percentage of women that are currently married, divorced, separated, or widowed.

**Table 5:** Men’s status in project countries

Variable	Countries						
	Bangladesh	India	Burkina Faso	Kenya	Tanzania	Mozambique	Uganda
Project implementers operating in country	BRAC, CARE	Landesa, IRRI	Helen Keller International	KickStart	KickStart	Land O’Lakes	Harvest Plus
Households headed by male (%)	89.0	85.1	91.7	64.2	75.2	64.7	70.8
Ever married (%)^∗^	63.7	68.0	59.5	53.2	58.5	65.5	63.7^b^
Median age at first marriage^+^	24.2	21.5	25.1^e^	24.8^+++^	23.6	24.4^++++^	21.9^++^
Median years of schooling	3.1	NR	NR	5.2^c^	6.3	3.7	5.3
Literate (%)	57.9	72.3	24.9	89.6	77.6	59.8	74.1
Currently employed (%)	98.8	85.7	97.8	86.7	85.9	82.8	91.6
No access to information (%)^a^	26.1	25.3^d^	33.1	8.2	23.9	31.8	13.2

*Source:* Authors. See Appendix for data sources.

*Notes:* All figures refer to rural population unless otherwise noted by an asterisk (∗). NR = “not reported.” + = ages 25–49. ++ = ages 25–54. +++ = ages 30–54. ++++ = ages 25–64. a = the percentage of men that do not read the newspaper, listen to the radio, or watch television at least once a week. b = this percentage includes living together, which comprises 15.11%. c = males age 6 and up. d = phrased differently: “percentage of men not exposed to any media.” e = men ages 30–59. l = the percentage of men that are currently married, divorced, separated, or widowed.

Some insights can be drawn from Table 4, Table 5 in regard to differences between men and women within the same country and also between women (and between men) in different countries. Most striking is the consistent pattern of lower levels of almost all indicators for women than for men within the same country. In all countries, men have higher levels of household headship, current employment, and access to information. Men are also, on average, older when they first marry and are less likely to have ever married. For all countries with the exception of Bangladesh, men have more years of schooling on average and a higher literacy rate. Bangladesh’s exception may be due to their extremely successful educational cash transfer program implemented in 1994, which some scholars have demonstrated has actually reversed the gender gap in schooling (Asadullah & Chaudhury, 2009).

With regard to differences between women in different countries, some patterns can be established by region. Women in South Asia are, on average, more likely to have ever married and also more likely to marry at an earlier age than women in Africa south of the Sahara. They are also generally less likely to be heads of households than women in Africa (with the exception of Burkina Faso, which showcases the lowest levels of female household headship of all of the countries). Even though the median age at first marriage is later in Africa than in South Asia, higher fertility rates are found across the African countries. No consistent regional trend is observed for involvement in decisionmaking or agreement with wife beating.

## 5 Gender, property rights, and changes in assets

### (a) Use, control, and ownership of assets in project households

For each project, quantitative data on assets were collected using survey modules covering physical assets and asset categories. Specific questions regarding the identity of the owner of the asset were included in each survey, though they were not always asked in the same way.^5^ Most projects collected data on land, livestock, and consumer durables, or agricultural assets. Survey questionnaires were often customized for the specific project contexts, for example, projects focused on crop production asked about specific pieces of farm machinery. Projects in South Asia asked about jewelry, which is very important for status as well as a store of wealth that can be liquidated to meet immediate needs, and is a particularly important asset for women (Quisumbing, Roy, Njuki, Tanvin, & Waithanji, 2013). The data collected provide important information on the types of assets that households have and how those assets are distributed within and across households. With a few exceptions, projects collected data on key assets, not the entire asset portfolio, and did not always collect information on the value of the assets. Therefore the data collected do not provide a full description of individual or households asset portfolios, nor do they permit aggregation across asset types to look at net effects.

To get a sense of asset ownership levels and how assets are distributed within households, we look at data from project beneficiaries and control groups on ownership of a four commonly collected assets or asset categories—land (Table 6), livestock (Table 7), and consumer and agricultural durables (Table 8).^6^ Ownership is divided into three categories: male-owned, female-owned, and jointly-owned. With few exceptions—consumer durables in HKI (Table 8), some livestock in BRAC and CARE endlines (Table 7)—the number, share, and/or value of male-owed assets is higher than that of female-owned assets for all assets and all projects. Rarely does the number or value of women’s assets reach half of that of men’s.

**Table 6:** Landownership by project beneficiaries at baseline and endline (selected projects)

Project implementer	Units		Baseline			Endline		
			Male-owned	Female-owned	Jointly-owned	Male-owned	Female-owned	Jointly-owned
BRAC	Value in taka	Treatment^a^	Not collected	Not collected	Not collected	33986.23	12773.81	501.1219
						(117394.10)	(66133.15)	(8065.18)
		Control^a^	Not collected	Not collected	Not collected	20232.5	10438.86	864.8547
						(8838.23)	(44139.17)	(21840.55)
CARE	Decimals^b^	Treatment	63.55	4.41	0.29	61.03	3.92	0.26
			(108.31)	(28.30)	(3.71)	(91.18)	(24.18)	(3.71)
		Control	62.34	3.48	.31	58.43	3.30	.32
			(102.67)	(19.97)	(3.83)	(87.00)	(20.66)	(3.68)
HarvestPlus	Acres	Treatment	1.94	0.12	0.83	1.96	0.18	0.80
			(3.72)	(0.57)	(5.77)	(2.62)	(0.99)	(2.48)
		Control	1.86	0.13	0.61	1.67	0.17	0.67
			(4.36)	(0.52)	(1.95)	(2.42)	(0.62)	(2.44)
Helen Keller International	Hectares	Treatment	3.2	1.4	Not collected	3.1	0.8	Not collected
			(3.1)	(5.4)		(3.9)	(1.7)	
		Control	3.1	1.2	Not collected	2.8	1.6	Not collected
			(2.8)	(1.8)		(1.9)	(8.2)	
Land O’Lakes	Acres	Treatment	3.17	.76	1.00	3.17	.76	1.00
			(4.07)	(1.50)	(2.65)	(4.07)	(1.20)	(2.65)
		Control	2.77	.53	.46	2.77	.53	.46
			(3.17)	(1.2)	(1.92)	(3.17)	(1.92)	(1.92)

*Source:* adapted from Quisumbing *et al.* (2015).

*Notes.* Table contains means. Numbers in parentheses are standard deviations. a = baseline data collected from the BRAC project was not disaggregated by sex of owner. b = 100 decimals = 1 acre.

**Table 7:** Livestock ownership by project beneficiaries at baseline and endline (selected projects)

Project Implementer	Units		Baseline			Endline		
			Male-owned	Female-owned	Jointly-owned	Male-owned	Female-owned	Jointly-owned
BRAC	Value, 2012 taka^a^	Treatment	Not collected^b^	Not collected	Not collected	1335.71	9932.28	1858.26
						(5603.82)	(13503.06)	(9043.28)
		Control	Not collected	Not collected	Not collected	461.52	1892.62	417.93
						(2900.96)	(8553.06)	(3066.42)
CARE	Cattle, value, 2008 taka	Treatment	18919.69	4677.95	13241.10	21867.37	5303.58	9699.02
			(30749.94)	(13915.35)	(30218.99)	(46748.58)	(26952.39)	(48012.05)
		Control	16455.07	4367.71	16760.82	22530.57	4896.59	9814.44
			(27724.48)	(12876.76)	(34734.04)	(45516.66)	(23510.62)	(42972.64)
	Goats, value, 2008 taka	Treatment	529.84	229.45	407.17	523.78	606.19	124.43
			(1543.20)	(981.95)	(1528.40)	(2218.87)	(1827.04)	(774.99)
		Control	457.93	206.67	486.60	447.10	625.62	106.43
			(1359.74)	(890.73)	(1624.19)	(1931.16)	(1711.65)	(724.45)
HarvestPlus	Share of value, 2007, thousand UGX	Treatment	0.51	0.24	0.25	0.51	0.24	0.26
			(0.44)	(0.35)	(0.42)	(0.43)	(0.34)	(0.42)
		Control	0.49	0.25	0.26	0.50	0.26	0.25
			(0.43)	(0.35)	(0.42)	(0.43)	(0.36)	(0.42)
Helen Keller International	Small animals (value in constant XOF)^c^	Treatment	123,617	26,319	Not collected	212,365	55,011	Not collected
			(157,316)	(48,251)		(262,249)	(74,706)	
		Control	139,499 (166,398)	29,034 (49,906)	Not collected	212,309 (262,952)	56,181 (76,944)	Not collected
	Large animals (value in constant XOF)	Treatment	370,695	6,463	Not collected	816,751	5,916	Not collected
			(495,489)	(52,024)		(1,283,962)	(42,398)	
		Control	425,789	12,444	Not collected	753,053	7,917	Not collected
			(512,365)	(71,783)		(1,049,704)	(54,489)	
Land O’Lakes	# of cattle	Treatment	3.08	.23	1.47	3.46	.20	1.53
			(5.83)	(1.24)	(3.26)	(6.23)	(1.43)	(3.22)
		Control	1.58 (4.23)	0.00 (0.00)	2.5 (6.85)	1.63 (3.34)	0.00 (0.00)	2.59 (6.85)

*Source:* adapted from Quisumbing *et al.* (2015).

*Notes.* Table contains means. Numbers in parentheses are standard deviations. a = includes cows, goats, chickens, horses, pigeons. b = baseline data collected from the BRAC project was not disaggregated by sex of owner. c = small animals include poultry and small ruminants, large livestock include cattle UGX = Ugandan shillings; XOF = West African CFA franc.

**Table 8:** Consumer and agricultural durables ownership by project beneficiaries at baseline and endline (selected projects)

Project Implementer	Units		Baseline			Endline		
			Male-owned	Female-owned	Jointly-owned	Male-owned	Female-owned	Jointly-owned
BRAC	Consumer durables (Value in 2012 taka)	Treatment	Not collected	Not collected	Not collected	6590.68	5018.10	2053.80
						(18460.80)	(8444.36)	(7987.99)
		Control	Not collected	Not collected	Not collected	3862.66	4310.24	1313.48
						(12382.82)	(10295.10)	(4414.18)
	Agricultural durables (Value in 2012 taka)	Treatment	Not collected	Not collected	Not collected	558.98 (2099.99)	345.36 (995.69)	189.18 (1765.93)
		Control	Not collected	Not collected	Not collected	195.69	193.74	97.91
						(1152.86)	(516.82)	(508.17)

CARE	Consumer durables (Value in 2008 taka)	Treatment	3954.87	611.68	3402.46	7,116.69	1,100.04	3,281.38
			(7160.79)	(2170.30)	(9661.16)	(12743.08)	(4045.94)	(7186.20)
		Control	4000.13	530.85	3384.04	7018.50	1062.42	3114.74
			(7579.46)	(1987.87)	(9444.18)	(13277.81)	(3613.22)	(6687.92)
	Agricultural durables (Value in 2008 taka)	Treatment	1544.79	49.43	944.29	2793.51	228.46	456.42
			(6313.35)	(587.34)	(3806.65)	(11225.06)	(4948.86)	(2143.29)
		Control	1596.52	43.83	1268.35	2475.44	165.76	488.21
			(7623.52)	(502.69)	(4559.82)	(9660.24)	(4165.64)	(2289.84)

HarvestPlus	Consumer durables (Share of value, 2007, thousand UGX)	Treatment	0.60	0.11	0.30	0.59	0.12	0.33
			(0.40)	(0.25)	(0.46)	(0.40)	(0.26)	(0.46)
		Control	0.58	0.12	0.30	0.60	0.12	0.24
			(0.40)	(0.27)	(0.46)	(0.39)	(0.26)	(0.42)
	Agricultural durables (Share of value, 2007, thousand UGX)	Treatment	0.47	0.11	0.42	0.50	0.12	0.38
			(0.46)	(0.25)	(0.49)	(0.46)	(0.26)	(0.48)
		Control	0.47	0.12	0.41	0.50	0.12	0.38
			(0.46)	(0.27)	(0.49)	(0.46)	(0.26)	(0.48)

Helen Keller International	Consumer durables (Value in constant XOF^a^)	Treatment	25,672	32,067	Not collected	25,680	38,277	Not collected
			(45,788)	(39,475)		(35,030)	(37,684)	
		Control	30,207	33,137	Not collected	25,892	38,370	Not collected
			(41,927)	(34,801)		(33,993)	(39,855)	
	Agricultural durables (Value in constant XOF^a^)	Treatment	23,395	1,537	Not collected	24,072	4,035	Not collected
			(47,395)	(3,232)		(36,406)	(9,747)	
		Control	23,241	1,853	Not collected	28,078	2,101	Not collected
			(35,524)	(3,903)		(66,709)	(7,864)	

Land O’Lakes	Consumer durables (Asset index)	Treatment	2.723	0.830	5.319	3.830	0.862	5.319
			(4.041)	(2.746)	(6.794)	(4.710)	(2.754)	(6.794)
		Control	2.947	0.211	6.211	3.211	0.211	6.211
			(7.656)	(0.713)	(4.650)	(7.685)	(0.713)	(4.650)
	Agricultural durables (Asset index)	Treatment	2.28	0.13	7.78	3.32	0.14	7.80
			(4.85)	(0.68)	(8.21)	(6.13)	(0.72)	(8.24)
		Control	2.56	0.16	5.76	3.53	.16	5.76
			(4.00)	(0.80)	(3.86)	(6.27)	(0.80)	(3.86)

*Source*: adapted from Quisumbing *et al.* (2015).

*Note*. Table contains means. Numbers in parentheses are standard deviations. UGX = Ugandan shilling; XOF = West African CFA franc. a = CFA francs are fixed to the euro in a ratio of 1 euro = 655.957 CFA francs or 1 CFA franc = 0.00152449 euros.

While men clearly own the majority of individually-owned assets, the data reveal a considerable amount of joint ownership. A significant share of household land is reported to be under joint ownership, especially in Africa. Including jointly-owned land significantly expands the amount of land over which women have ownership rights. Joint ownership is even more important for livestock and consumer durables, with the share of jointly-owned animals close to or even exceeding that owned by men individually and always greater than that owned by women individually.

One way to interpret jointness is that two or more individuals share rights to a single asset and make joint decisions. Another possibility is that individuals have different rights over the same asset. These types of jointness may not be mutually exclusive, though in many gender-asset survey modules (such as the ones used to collect the data in Table 6, Table 7, Table 8) this condition is often imposed to ensure that the sum of the number of assets under each ownership category adds up to the number of assets owned by the household (a common check on data quality and accuracy).

All of the qualitative and some of the quantitative studies (for example, Das et al., 2013, Paris et al., 2015, Quisumbing et al., 2013, Roy et al., 2015) looked deeper into jointness and explored different rights that men and women, individual or jointly, have over different types of assets. In general, where men and women have different rights to the same asset, men tend to have more and stronger rights than women. For example, a wife often has the right to use her husband’s land (for example, Gilligan et al., 2013, van den Bold et al., 2015). Women control milk for home consumption, but men control income from milk sales to collection centers (Johnson, Njuki, Waithanji, Nhambeto, Rogers & Kruger, 2015). Woman can use a pump but not loan it out to others without permission (Njuki, Waithanji, Sakwa, Kariuki, Mukewa, & Ngige, 2014).

The qualitative analyses provided many examples where husbands and wives discussed what to do and made decisions together. However, where they could not agree, it was almost always the man who had the final say. In most cases, men felt they owned all household assets by virtue of their being heads of households. As one respondent in a focus group discussion (FGD) for the KickStart project said, “Men have the right to sell all assets, even those owned by women” (Njuki *et al.*, 2014). One exception was when a woman’s name was on a title to land, so that the land could not be sold without her permission. And in some cases, a man’s decision to sell an asset without a woman’s permission may not be final. In the BRAC project, when a man sold an asset without his wife’s permission, she was able to appeal to a project authority for the return of the asset (Das *et al.*, 2013). These cases were possible because women were aware of their rights and had access to a means of defending them that they were willing and able to use.

These results suggest that jointness is the rule rather than the exception and is likely to matter even for assets that were reported to be individually owned. More research is needed on how to define, measure, and understand the importance of jointness. Drawing on the literature on collective action among households for natural resource management, Doss and Meinzen-Dick (2015) suggest directions for research on what enables households to reach cooperative outcomes, which can include increases in the stock of joint assets, and what kinds of policy interventions (such as joint registration of land) can support cooperation within households as well as communities. In a project context, the importance of jointness in asset ownership may depend on the project’s objective. For dissemination of annual cropping technologies, use rights may be sufficient for uptake. Where longer-term investments are required (such as tree planting), control rights are likely to be needed to ensure that people can realize the benefits of their investments over time. Knowing who has control over the outputs generated from productive assets (such as milk from a cow, crops from land) is important in understanding who is likely to benefit from that asset’s use, and therefore have an incentive to invest in obtaining and maintaining it. Some evidence on these issues is presented in the following sections.

Open-ended questions about who owned what assets and which assets were important for men and women yielded interesting information about gender differences in perceptions and priorities. The differences in perception can offer valuable insights into how projects work. For example, in the Land O’Lakes project, cows were clearly given to household heads, usually men, yet many spouses felt they had some ownership rights due to the importance of their contribution to taking care of the cow and to the household dairy business (Johnson *et al.*, 2015).

FGDs also identified assets that researchers had not previously considered. In several cases FGDs identified things like access to government programs, jobs, or nongovernmental organization (NGO) trainings as assets. In FGDs in Tanzania, men referred to women as assets that they (men) owned! A lesson from these experiences is that, as in quantitative surveys, it can be useful to provide some guidelines or examples to guide the discussion on a topic as complex as asset ownership. However, where there is opportunity to discuss with respondents, interviewers can probe further to understand the reasons behind why something is identified as an asset. In the FGDs conducted as part of the IRRI evaluation, people identified expensive clothes as an important asset (Paris *et al.*, 2015). Having good clothes enables people to attend meetings and other social events at which they can build social capital (Das et al., 2013, Paris et al., 2015).

### (b) How did projects change asset ownership?

All project evaluations document increases in some types of assets among target households. Some projects collected asset data on a number of different types of assets and were able to assess impacts on both target and non-target assets. Among the projects that completed quantitative impact evaluations, all of them, whether or not they transferred assets, contributed to increases in levels of some or all measured assets in beneficiary relative to control households (Table 9).^7^ In four projects, quantitative analysis found that the project significantly increased women’s assets, usually the assets targeted by the project, and strengthened their rights to those assets. Landesa’s *Nijo Griha*, *Nijo Bhumi* program in West Bengal increased women’s perceived tenure security as compared with control households where women’s names were not on titles (Santos, Fletschner, Savath, & Peterman, 2014). HKI’s E-HFP in Burkina Faso increased the value of agricultural assets of women in intervention villages relative to women in control villages (van den Bold *et al.*, 2015). In BRAC’s TUP project, women’s sole and joint ownership of targeted assets (cattle, goats, poultry) increased compared with that of women in nonparticipant households (Das et al., 2013, Roy et al., 2015). The BRAC study also looked at women’s specific rights (to rent, sell, and control product and income) and found that, in general, the program increased women’s use rights, but not ownership rights, to agricultural assets. Finally, the CARE SDVC project in Bangladesh did not increase women’s individually-owned assets but did increase jointly-owned nonagricultural productive assets (Quisumbing *et al.*, 2013).

**Table 9:** Summary of key project impacts on assets, as measured in quantitative impact assessment using experimental or quasi-experimental methods

Implementer	Variable definition	Estimation method	Impact on asset outcome, relative to control			
			Women	Men	Jointly-owned	Household-level or other
Landesa	Woman reports that her household will have the same or more access and control over the plot in five years (mean 0.84, se 0.01)	Propensity weighted regression				0.18^∗∗∗^
						(0.01)
	Woman reports that she will have the same or more access and control over the plot in five years (mean 0.86, se 0.01)		0.17^∗∗∗^			
			(0.01)			

BRAC	Value of land owned, 2012 taka	Single-difference estimates	1,808	11,292^∗∗∗^	−56	13,676^∗∗∗^
			(1,630)	(2,670)	(386)	(4,278)
	Value of livestock, 2012 taka		9,090^∗∗∗^	942^∗∗∗^	1,511^∗∗∗^	11,703^∗∗∗^
			(401)	(148)	(192)	(410)
	Value of agricultural durables, 2012 taka		173^∗∗∗^	375^∗∗∗^	98^∗∗∗^	725^∗∗∗^
			(25)	(48)	(37)	(82)
	Value of household durables, 2012 taka		767^∗∗∗^	2,437^∗∗∗^	704^∗∗∗^	4,894^∗∗∗^
			(295)	(388)	(209)	(785)

CARE	Cattle, value in 2008 taka	Propensity-weighted ANCOVA regressions	603.72	–3,796.39	1,911.73	–431.16
			(1,518.05)	(9,757.10)	(5,701.45)	(3,107.94)
	Goats, value in 2008 taka		–62.99	199.59	51.148	320.33^∗^
			(223.20)	(134.02)	(67.639)	(191.86)
	Poultry, value in 2008 taka		0.52	23.62	–14.65	23.08
			(89.61)	(78.92)	(34.57)	(120.46)
	Agricultural assets, value in 2008 taka		183.40	940.33	–95.32	1,303.25^∗^
			(167.89)	(616.81)	(441.57)	(690.24)
	Nonagricultural productive assets, value in 2008 taka		60.19	253.68	127.74^∗∗^	452.58^∗^
			(51.37)	(231.68)	(58.44)^b^	(252.50)^c^
	Consumer durables, value in 2008 taka		70.95	347.58	485.54	4,874.67
			(328.39)	(1,213.80)	(852.04)	(4,401.01)
	Owned land, area in decimals		0.48	6.92	–0.18	7.65
			(0.92)	(7.95)	(0.43)	(11.30)

Helen Keller International	Value of household durables	Double-difference estimation method	65.62	2,352		
			(3,398)	(4,181)		
	Value of agricultural assets		2,133^∗∗∗^	−3,388		
			(592)	(3,499)		
	Value of small animals		1,979	29,352		
			(6,418)	(21,437)		
	Land cultivated (hectares)		−0.45	0.27		
			(0.41)	(0.24)		
	Hemoglobin concentration, children 3–12.9 months at baseline, health committee treatment^d^					0.51^∗^
						(0.27)
	Hemoglobin concentration, children 3–5.9 months at baseline, health committee treatment^e^					0.76^∗∗^
						(0.33)

HarvestPlus	Vitamin A (μg RAE/day), children 6–35 months of age (more intensive extension model)^a,f^	Double-difference estimation method				297^∗∗^
						(51)
	Vitamin A (μg RAE/day), children 6–35 months of age (less extensive extension model)^a,g^					229^∗∗^
						(52)
	Vitamin A (μg RAE/day), women (more intensive extension method)^a,h^		763^∗∗∗^			
			(69)			
	Vitamin A (μg RAE/day), women (less intensive extension method)^a,i^		591^∗∗∗^			
			(76)			

*Sources:* Landesa (Santos *et al.*, 2014); BRAC (Roy *et al.*, 2015); CARE (Quisumbing *et al.*, 2013); Helen Keller International (Olney et al., 2015, van den Bold et al., 2015); HarvestPlus (Hotz *et al.*, 2012).

*Notes.* Variables are as defined in tables 6-8 unless otherwise noted. Absolute value of standard errors in parentheses. Test statistics are t statistics. ^∗^ = significant at the 10% level, ^∗∗^ = significant at the 5% level, ^∗∗∗^ = significant at the 1% level. a = Adjusted vitamin A intake levels reported; for details see Hotz *et al.* (2012)Table 3. b = mean value at endline for jointly-owned assets: treatment (190.02 (2,586.72); control: 134.86 (2,178.41). c = mean value at endline for household assets: treatment 952.37 (5511.73); control: 790.04 (4685.30). d = mean value at endline treatment: 9.89 (1.43); control: 9.68 (1.42). e = mean value at endline treatment: 9.87 (1.56); control: 9.58 (1.38). f = Mean value at endline treatment: 518 (21) control: 258 (20). g = mean value at endline treatment: 414 (16) control: 258 (20). h = mean value at endline treatment: 1270 (60) control: 667 (32). i = mean value at endline treatment: 1130 (80) control: 667 (32).

Three of the four projects that increased women’s assets (Landesa, HKI, and BRAC) distributed assets directly to women and took steps to ensure that women maintained control of the transferred assets. The steps taken—putting a women’s name on the land title, supporting women to reclaim assets that were taken from them, influencing gender norms about asset ownership—varied but all had the effect of supporting the initial asset transfer. In the case of CARE, the project did not distribute assets so the increase in joint ownership of nonagricultural productive assets came about because dairy incomes were reinvested in other types of assets, diversifying the asset portfolio. Respondents implicitly recognized women’s contribution to that increased investment by considering these assets as joint assets.

Men in participant households also increased their assets through the projects (Table 9). The BRAC evaluation found that, relative to the control group, while women’s ownership of program assets grew, men’s sole ownership of all other assets grew (Das et al., 2013, Roy et al., 2015). CARE reported significant increases in household-level assets (goats, agricultural productive assets, non-agricultural productive assets) relative to a control group of households in the same villages that did not participate in the dairy value chain program. These increases were driven largely by increases in male-owned assets, although the increase in male assets is not significantly different relative to the control group.

Only in HKI was there any evidence of women closing the gender asset gap. Women’s value of agricultural assets in intervention villages increased, whereas men’s decreased (van den Bold *et al.*, 2015). There was no impact of the project on the area of land cultivated by either men or women, although qualitative work indicates that gender norms became more favorable toward women’s landownership in treatment as compared with control areas. While HKI did not explicitly seek to influence norms, the project recognized that empowering women is crucial to achieving nutrition objectives. In addition to distributing inputs and providing training to women beneficiaries of the program, the project also negotiated with the community for land on which women could establish a village model farm (VMF). In past HKI projects in other countries, VMFs were often run by male, farmer leaders. While the male farmers were able to operate successful model farms, the extent to which their example reached the ultimate target farmers, often poor women, was found to be limited (Hillenbrand, 2010). Therefore, a new approach was taken in which the practices promoted by the project were modeled on communal farms run by women themselves. The process of establishing the communal farm raised visibility and engaged the broader community in the project. Some of those who reported changing their opinion about women owning land attributed the change to the project and to what they observed in the VMF (van den Bold *et al.*, 2015).

Taken together, these results show that while it is possible to increase women’s control and ownership of assets, it is not easy or automatic, even in projects that transfer assets to women. Some common barriers that projects faced include: resistance or uncooperative attitudes of local government officials or even program staff toward women asset owners; deep-seated cultural norms that view particular types of assets as men’s assets, such that even if a program targets women, men still maintain control of those assets and make major decisions regarding them; and possible dilution of the net effects of the asset transfer as proceeds are reinvested in assets owned by men.

Recognizing joint ownership—not only in how assets are measured but also in how asset transfers are designed in projects that target households—could broaden the scope for change. The BRAC and IRRI evaluations asked not only about asset ownership but about what things women could and could not do with specific assets. Basing both analysis and program design (participation requirements, asset transfer modalities) on a more detailed understanding of what assets are expected to enable individuals and households to do may be more effective than relying on a binary definition of ownership.

Perhaps most sobering is the finding that the gender asset gaps are rarely narrowed and may be increased as a result of agricultural development projects. Given the gender norms that govern asset ownership, to the extent that project benefits get reinvested in assets, those assets are likely to be controlled primarily or exclusively by men. Explicit steps appear to be necessary to increase the chance that women will maintain and accumulate assets, including efforts to influence the norms around the acceptability of women having control of assets, either individually or jointly with others in the household.

## 6 Assets to livelihood strategies

In its most general sense, the conceptual framework posits that the gendered distribution of assets within a household influences uptake of technologies and livelihood strategies by household members. Several projects provide evidence to support this hypothesis.

The evaluation of the Landesa microtitling project in Odisha looked at the role of gendered asset ownership in the adoption of livelihood strategies (Savath, Fletschner, Peterman, and Santos, 2014) and found that men’s and women’s education and ownership of land and other (nonproductive) assets were important in enabling households to adopt preferred livelihood strategies, defined as those that offered higher incomes and greater food security. As compared with the least preferred strategy of agricultural wage labor, the more desirable livelihood strategies combine off-farm work, either for a wage or as a self-employed businessperson, with farming. These strategies are more likely to be adopted where women own a larger share of household assets. Men’s but not women’s education is important for wage labor; however, women’s education is positively associated with self-employment.

The HarvestPlus project in Uganda found that orange sweet potato (OSP) was more likely to be adopted on plots of land that were jointly-owned by men and women but where women played the leading role in decisionmaking (Gilligan *et al.*, 2013). Conversely, the probability of adopting OSP was lowest for parcels controlled exclusively by men.

In projects that were able to increase women’s assets, we can look at the impact of that increase on women’s participation in decisionmaking related to adoption of technologies and strategies, and also at its influence on the outcomes of those (often household-level) decisions. The Landesa evaluation found that having a woman’s name on the title was significantly associated with an increase in her reported participation in decisions about the purchase of productive assets and use of agricultural land (Santos *et al.*, 2014). It was also positively correlated with the share of household land over which the woman had an influence. The study also found significant program impacts on the outcomes of household decisions such as taking agriculture-related loans and investing in agricultural inputs (use of fertilizer, seeds, hired equipment); however, having the woman’s name on the title was not a significant predictor of these decisions.

HKI found high levels of adoption of home gardens by program households as compared with households in control communities (van den Bold *et al.*, 2015). Women were the main decisionmakers about vegetables, and this decisionmaking ability increased over the study period. Women were also primarily responsible for the chickens. Women increased their decisionmaking power over goats as compared with women in control communities; however, men continued to have primary responsibility for the goats even in intervention communities.

Separate evaluations of the BRAC program (Bandiera et al., 2013, Krishna et al., 2012) found that it was very successful in contributing to outcomes such as households’ overall food expenditure, rates of self-employment, and labor force participation, as well as household-level ownership of productive assets. The GAAP evaluation found that the project contributed significantly to a major shift toward women working inside the home in program households as compared with women in a control group; 17% more women worked inside the home and 8% fewer worked outside the home (Das *et al.*, 2013). This reduction of women’s mobility was not surprising given that the assets the program provided to women, especially cattle, require care at home; that social norms in Bangladesh favor women’s seclusion; and that poor women who worked outside the home were typically in low-status occupations like domestic work and agricultural wage labor and were often harassed.

Although the CARE project did not introduce dairy as a new livelihood strategy, it did intend to improve the profitability of that strategy and to involve women to a greater extent in stages of the value chain, for example, as livestock health workers and artificial insemination providers, where they were traditionally underrepresented. The project was able to increase women’s jointly-owned assets, but these results did not translate into greater participation in decisions related to buying, selling, or leasing of cows or to dairy-related expenses—financial decisions remained the husbands’ domain. In terms of specific decisions, however, the program led to significant increases in women’s decisionmaking in feeding cows (10%) and on where to purchase inputs (4%) but had no effect on other decisions related to vaccinations or artificial inseminations, activities on which women had also received training (Quisumbing *et al.*, 2013).

Similar to BRAC, the CARE project reduced the proportion of women working for pay, mainly because domestic responsibilities, largely related to livestock, increased. This does not appear to be because of husband’s objections: analysis of program impacts on mobility suggest that, although the magnitudes of the effects are modest, the project may have caused a slight gain in acceptability of women leaving the home, even if only through more joint decisions and through an expanded set of conditions under which leaving the home is acceptable (Quisumbing *et al.*, 2013). This is consistent with women’s claims that the decrease in women working outside the home was the women’s own choice. The program did improve attitudes toward women’s mobility, particularly with respect to her ability to go to locations where she could access livestock-related services.

While the impact evaluation did not find significant impacts on women’s asset ownership, both the qualitative and the quantitative data in the Land O’Lakes study suggest that women are claiming some ownership rights to the transferred dairy cows (Johnson et al., 2015, Quisumbing et al., 2015). Both male and female FGD participants report that women play an active role in dairying. Prior to the project women were not involved in cattle keeping. Perhaps as a result, project staff did not initially include women in the dairy project, but they observed that cows that had been transferred to beneficiary households were not doing well. Further investigation by project staff revealed that cows were not being adequately fed and cared for and that a main reason for this was that men were assuming that women would take care of them even though they had neither been involved in the project nor received training on the needs of Jersey cows. As a result of this finding, the project began involving women in project activities. This anecdote was part of the motivation for the Land O’Lakes project to join GAAP to better understand gender dynamics and impacts.

The KickStart project enabled households to take up irrigation, a livelihood strategy that reduced risk and increased income. Few women purchased pumps (only 6% of pump sales in Tanzania and 18% in Kenya were by women), and the findings of the qualitative analysis conclude that while there is some joint decisionmaking, the “main decisions on crop choice were in the hands of men irrespective of whether women owned the pumps or not” (Njuki *et al.*, 2014, p. 21).

These results provide some evidence for the hypothesis that strengthening women’s assets will increase their role in decisionmaking about livelihood strategies. The evidence is stronger for participation in decisionmaking than in influencing the outcomes of decisions, though the former may be a necessary first step toward the latter. Some of the strategies that women choose to adopt may not be the ones that appear to an outsider to be most desirable, but they may well be optimal given the constraints and trade offs women face in the contexts in which they live.

## 7 Impacts on full income

### (a) Impacts on household income

According to the conceptual framework, livelihood strategies result in full income, which is defined as “the total value of products and services produced by the household members, some of which are consumed directly and others which are sold for cash or traded for other goods or services” (Meinzen-Dick *et al.*, 2011, p. 12). The concept of full income also includes the leisure time of household members. Because it is more likely for women’s time to be devoted to nonmarket or reproductive activities—including growing food consumed at home, caring for children, and caring for the ill—measures of income that do not take into account the value of time will tend to underestimate women’s contribution and overestimate the benefits of activities that increase both cash income and workloads (Meinzen-Dick et al., 2011, Quisumbing et al., 2015). Some market-oriented agricultural interventions seek explicitly to increase cash income, but all interventions seek to increase full income, whether explicitly or implicitly. The hypothesis to be explored in this section is whether increasing women’s control over assets will influence their control over the income.

Before looking at income control, we first look at changes in income levels. Few projects collected detailed data on agricultural inputs, outputs, sales, or prices, and none conducted productivity analysis as part of GAAP. In general, however, projects reported that where assets were transferred and where improved technologies were adopted, production of target crops and livestock products increased. Most households reported increases in income, but they were not always statistically significant as compared with control groups.

All of the evaluations of asset-transfer projects reported that the transfer of assets had impacts on women’s time. All projects that transferred livestock—BRAC, Land O’Lakes, and HKI—found that caring for livestock, especially improved or exotic breeds that tend to have greater nutrition and health needs, led to an increase in demand for women’s time (Quisumbing *et al.*, 2015). It is important to note, however, that these new livelihood strategies increased demand for time of other household members as well, including men and children. The Land O’Lakes study reported that although the greatest increase in time spent on dairy was for women’s time, labor increased for all household members and men provided the largest amount of total labor for dairy production (Johnson *et al.*, 2015).

To understand the impact of these increased time burdens, we need to know what household members were doing with their time before the projects. The CARE evaluation looked at changes in time spent by household members in a range of activities and found that women in program households spent more time on dairy and less on childcare (feeding and general care) than control households in the same communities (Quisumbing *et al.*, 2013). This could be a cause for concern because the time women spend on childcare is a determinant of child nutritional outcomes (Herforth & Harris, 2014). Findings from the BRAC evaluation state that some women complain about workloads associated with program assets and said that other family members had to help with care of livestock, especially cows (Das *et al.*, 2013). Nonetheless, they prefer their current situation to the previous situation of not having livestock and working outside the home.

The two projects that promoted machinery—KickStart and IRRI—found slightly different results. While the KickStart pumps sometimes required additional labor from women, it was also reported that women reduced time spent fetching water (Njuki *et al.*, 2014). IRRI promoted labor-saving technologies that had the potential to displace female agricultural labor. In better-off, high-caste households, adoption of these technologies was likely to lead to more leisure time for women; but women in poorer, landless households would lose an important source of income (Paris *et al.*, 2015).

Although some exceptions are clear, these results suggest that women are aware of and willing to make sacrifices in terms of time because they value the benefits of the project. Projects should monitor their impacts on time, and where there is evidence that dedication of time to project activities is having negative impacts on other outcomes that households are not aware of or not considering, targeted activities could be undertaken to mitigate possible negative impacts.

### (b) Impacts on control of income

Based on the conceptual framework, we would expect that greater control over productive assets and contribution to production processes would translate into greater control over the product or of income generated from the asset. In practice, support for this hypothesis was mixed.

When agricultural output was used for home consumption, women were more likely to report that they had some or full control over how it was used. Where output was sold, women’s involvement in the decision to sell and their control over the income was limited, even where they had some ownership rights to the underlying assets. HKI was primarily a nutrition-focused project, so much of the intervention involved supporting women to grow nutritious foods and prepare and serve them to their families. This program was found to significantly improve several child nutrition outcomes, including diet quality, wasting (marginal), diarrhea, hemoglobin, and anemia, especially among the youngest children (Olney, Pedehombga, Ruel, & Dillon, 2015). Income was a lesser objective, reflected by the fact that quantitative income data were not collected, but women reported that to the extent that they received income from vegetables and chickens, they were able to maintain control of it (van den Bold *et al.*, 2015). Men controlled income from goats.

The evaluation of Landesa’s project in West Bengal did not report impacts on income but did report that women’s participation in the decision to sell produce from the plot is positively and significantly related to whether her name is on the land title (Santos *et al.*, 2014). Although it did not (yet) find impact of the program on household food security, Landesa’s West Bengal study did find that where the woman’s name was on the land title, she was more likely to participate in household food purchase and consumption decisions.

Typical of the pattern of many dairy development projects, the Land O’Lakes study found that men controlled the morning milk, which is greater in quantity than the evening milk and is usually sold to the milk collection center. Women controlled the evening milk, which is split between calves, household consumption, and sale to neighbors (Johnson *et al.*, 2015). To the extent that they derive income from local sale of milk, women are able to control it, but the main cash income from the milk is from the morning milk and is controlled by the men.

The CARE project did not increase women’s decisionmaking power with respect to use of household income for food, house repairs, or health (Quisumbing *et al.*, 2013). It did, however, increase women’s control of money to buy food, clothes, medicines, and cosmetics for themselves.

In the KickStart project women did not have input into decisionmaking related to pump use (Njuki *et al.*, 2014). The type of crop irrigated did, however, influence income control by women, with women more likely to control income from irrigated leafy vegetables compared with that from other crops.

The BRAC project appears to have reduced control by women over income and decisionmaking about income. The evaluation found that compared with control households, beneficiary households had a reduced proportion of women who worked outside the home and kept all or any of the money (Das *et al.*, 2013). The program also reduced the percentage of households in which a woman decides alone what to do with income and an increased percentage of households where women and their husbands decide jointly what to do with the money she earns.

## 8 Impact on women’s and households’ welfare

Given their relatively limited control over the outputs and income that result from the assets and livelihood strategies promoted by the projects, what motivates women to invest time and effort in these projects? The conceptual framework suggests several explanations. One is that while women do not necessarily control the specific output or income from project assets or new agricultural technologies, they believe that the increases in food production or income at the household level will be used in ways that are consistent with their own preferences and will improve their own welfare and that of their families. A second explanation is that women perceive intangible benefits from participation in the projects. The studies found support for both explanations, which are not mutually exclusive.

In both Bangladesh projects (BRAC and CARE), women mentioned that they valued being able to contribute to the maintenance of their households. Participants in BRAC specifically mentioned the improved social standing that the increased income from the cow made possible—it enabled them to purchase saris, for example, so that they would no longer be ashamed to appear in public.

In two projects, one of the important benefits that women specifically associate with the projects is an increase in household cooperation and harmony. In the Land O’Lakes project, this took the form of families working together in a shared livelihood strategy of dairying in which husbands recognized wives’ expertise, gained through training and experience, and sought their advice (Johnson *et al.*, 2015). In KickStart households, the requirement for two people to operate the pump meant husbands and wives spent more time working together on the farm (Njuki *et al.*, 2014).

Several evaluations mentioned examples of changes in norms related to women’s roles in agriculture, in their households, and in society. The HKI evaluation, as described above, looked specifically at whether the project had an impact on individuals’ attitudes toward women’s land use and ownership. When asked if their attitudes regarding whether women were capable of using land and should be allowed to own land had changed in the last two years, respondents in program communities were significantly more likely to report changes in attitudes that favored women’s access to and control over land than respondents in control communities (van den Bold *et al.*, 2015).

The CARE project had a positive impact on women’s mobility (Quisumbing *et al.*, 2013). Compared with control households, the CARE project participants are more likely to have a say (together with their husbands) about whether they can go alone to visit friends outside the community, go to the market, or go to the cinema. Program participants are also more likely to have a say in decisions about whether they can attend NGO trainings. Not all changes associated with increased mobility and involvements in value chains were positive, however. The CARE project found that in some cases, women’s increasing involvement in value chain activities was contributing to increases in gender-based violence in dairy value chains. In response, according to the team leader, the project implemented a community-based intervention to raise awareness among men and engage them in helping to address the problem (Nurul Amin Siddiquee, personal communication, 2011).

Finally, both projects with explicit nutrition objectives, HarvestPlus and HKI, succeeded in improving diets and nutritional status of women and children (Hotz et al., 2012, Olney et al., 2015). These changes, if sustained, could contribute to improvements in future outcomes related to education, income, and health, reducing long-term inequities (Hoddinott, Rosegrant, & Torero, 2012).

Taken together these findings suggest that women value benefits at the household level and that they perceive many intangible benefits from participating in the projects, some of which could be related to changes in the broader social context, including in gender norms. If this is the case, it could be an important area for future research and for future emphasis in projects, because many of the norms around gender roles and women’s ownership of assets seem to prevent them from sharing more directly in the benefits of projects.

## 9 Conclusions

The studies in the GAAP portfolio show the myriad ways in which use, control, and ownership of a wide range of assets affect the ability of men and women to benefit from agricultural interventions. All projects were associated with increases in specific assets or asset types at the household level, but only half were able to increase women’s control or ownership of assets, and of those, only one, HKI’s Enhanced Household Food Production project in Burkina Faso, seems to have contributed to a reduction in the gender-asset gap. Many projects increased women’s income; however, when compared with changes in income at the household level and for male household members, it is clear that women find it difficult to increase their relative control over income from projects.

The findings also suggest that greater recognition of the importance of assets, and attention to issues of gender and asset ownership in project design and implementation as well as evaluation, could improve the ability of projects to benefit women.^8^ Reframing the gender asset gap with a greater emphasis on jointness as a way to increase women’s control of assets is a potential avenue that deserves further study. Also promising is the evidence that some projects (CARE, Land O’Lakes, HKI) may have influenced underlying household and community attitudes about women’s work, participation in decisonmaking, and capacity to use and control assets. While these were not explicit objectives of the projects, the changes suggest the potential to influence social and gender norms in ways that could lead to greater empowerment for women. Even where impact evaluations did not find significant asset or income benefits for women, qualitative and quantitative analysis identified many tangible and intangible ways that interventions improved women’s lives and welfare. Looking at a broader range of benefits—especially using mixed-method approaches—provides valuable insights into how projects work, how men and women participants experience their benefits and costs, and how this information can be used in project design and in the identification of indicators with which to monitor project outcomes.

The GAAP projects also made important methodological contributions to the study of gender and assets in developing-country contexts. Two of the most important were to demonstrate that collecting sex-disaggregated asset data is possible and feasible in a project context, and that asset measures are sensitive to change within development project time frames, typically three to five years, even in projects that do not transfer assets. The lessons learned in GAAP on how to understand and measure assets and property rights over assets, both quantitatively and qualitatively, have been widely shared and are available in a toolkit.^9^ The GAAP experiences were an important contribution to the development of the Women’s Empowerment in Agriculture Index (Alkire, Meinzen-Dick, Peterman, Quisumbing, Seymour, & Vaz, 2013),^10^ a tool whose development and widespread use is evidence of the growing recognition of the importance of considering measures of women’s empowerment, including increased control and ownership of assets, as outcomes to which agricultural development projects should be expected to contribute.

In most cases, the evaluations capture the short-term impacts of projects since the end lines were done shortly after project completion. In Landesa, the project had not yet finished so additional impacts are to be expected. BRAC was the second phase of a project (phase one ran from 2002 to 2006) and several other projects (HKI, CARE, Land O’Lakes) will have second phases. In most cases the second phases will not be in the same locations as the initial phases, however the lessons in terms of implementation that were learned in Phase 1 could be applied to Phase 2, potentially enhancing impacts. Looking at longer-term impacts should be a priority for future work in this area.

## Appendix A Data Sources

### A.1 Data sources for Table 4

Data for Bangladesh come from DHS Bangladesh Country Report 2011 (NIPORT, 2013) and refer to ever-married women ages 15–49 unless otherwise noted, except for statistic on land, which comes from FAO Gender and Land Rights Database and is drawn from the Agricultural Sample Survey 2005 (FAO, 2010). Data for Burkina Faso come from DHS Burkina Faso Country Report 2010 (INSD, 2012) and refer to women ages 15–49 unless otherwise noted, except for statistic on land, which comes from FAO Gender and Land Rights Database and is drawn from the Agricultural Census 1993. Data for India come from India National Family Health Survey Report 2005–2006 (IIPS, 2007) and refer to women ages 15–49, unless otherwise noted, except for statistic on land, which comes from FAO Gender and Land Rights Database and is drawn from the Census of Agriculture 2000–2001. Data for Kenya come from DHS Kenya Country Report 2008–2009 (KNBS, 2010) and refer to women ages 15–49. Data for Tanzania come from DHS Tanzania Country Report 2010 (NBS, 2011) and refer to women ages 15–49, unless otherwise noted, except for statistic on land, which comes from FAO Gender and Land Rights Database and is drawn from the Tanzania Agricultural Census 2002–2003. Data for Mozambique come from DHS Mozambique Country Report 2011 (MISAU (Ministerio da Saude), INE (Instituto Nacional de Estatística), & ICF International, 2011) and refer to women ages 15–49, unless otherwise noted, except for statistic on land, which comes from FAO Gender and Land Rights Database and is drawn from the Mozambique Agricultural Census 1999–2000. Data for Uganda come from DHS Uganda Country Report 2011 (UBOS, 2012) and refer to women ages 15–49, unless otherwise noted, except for statistic on land, which comes from FAO Gender and Land Rights Database and is drawn from the Report on the Uganda National Census of Agriculture and Livestock 1990–1991.

### A.2 Data sources for Table 5

Data for Bangladesh come from DHS Bangladesh Country Report 2011 (NIPORT, 2013) and refer to ever-married men ages 15–49 unless otherwise noted. Data for Burkina Faso come from DHS Burkina Faso Report 2010 (INSD, 2012) and refer to men ages 15–59 unless otherwise noted. Data for India come from India National Family Health Survey Report 2005–2006 (IIPS, 2007) and refer to men ages 15–54 unless otherwise noted. Data for Kenya come from DHS Kenya Country Report 2008–2009 (KNBS, 2010) and refer to men ages 15–54 unless otherwise noted. Data for Tanzania come from DHS Tanzania Country Report (NBS, 2011) and refer to men ages 15–49 unless otherwise noted. Data for Mozambique come from DHS Mozambique Country Report 2011 (MISAU *et al.*, 2011) and refer to men ages 15–49 unless otherwise noted. Data for Uganda come from DHS Uganda Country Report 2011 (UBOS, 2012) and refer to men ages 15–54 unless otherwise noted.

## Footnotes

1 For a more detailed explanation of the GAAP conceptual framework, including implications for project design, research, and evaluation, see Meinzen-Dick *et al.* (2011).

2 When we use the word *assets*, it is implicit that we are referring to their use, control, ownership, or any combination of these.

3 For more information on GAAP, including research and capacity-building outputs, see the project website: http://gaap.ifpri.info/.

4 For convenience we refer to projects by the name of the implementing organization; however, we are referring only to the actions and results associated with the specific projects included in the GAAP portfolio.

5 For more information on the methodology used to collect asset data, see the GAAP toolkit at http://gaap.ifpri.info/integrating-qual-and-quant/.

6 Table 6, Table 7, Table 8 report asset levels at baseline and endline with no attempt to attribute change to the project. See Table 9 for summary of the changes in assets and other development outcomes that can be attributed to the project interventions.

7 Not all projects collected sex-disaggregated assets data at baseline, and some projects only collected information on a subset of key assets. The BRAC baseline survey, for example, did not collect sex-disaggregated assets data, so comparisons are single-difference comparisons. However, because the program was randomized, our single-difference estimates are unbiased estimates of program impact. Some of the projects also estimated impacts only on those assets that were the focus of their project (e.g., Landesa and HarvestPlus focused on land rights and nutritional status, respectively).

8 http://gaap.ifpri.info/technical-guide/.

9 http://gaap.ifpri.info/files/2010/12/GAAP_Toolkit_Update_FINAL.pdf.

10 http://www.ifpri.org/book-9075/ourwork/program/weai-resource-center.
